# Quantitative evaluation of fungi of the genus *Candida* in the feces of adult patients with type 1 and 2 diabetes - a pilot study

**DOI:** 10.1186/s13099-014-0043-z

**Published:** 2014-10-15

**Authors:** Tomasz Gosiewski, Dominika Salamon, Magdalena Szopa, Agnieszka Sroka, Maciej T Malecki, Malgorzata Bulanda

**Affiliations:** Department of Microbiology, Jagiellonian University Medical College, 18 Czysta St, 31-121 Krakow, Poland; Department of Metabolic Diseases, Jagiellonian University Medical College, 15 Kopernika Street, 21-501 Krakow, Poland; University Hospital, Krakow, Poland

**Keywords:** *Candida*, Feces, Serum lipids, T1DM, T2DM

## Abstract

**Background:**

Gastrointestinal tract microbiota, particularly bacterial microflora, seem to have a different qualitative and quantitative composition in both type 1 (T1DM) and type 2 diabetes (T2DM) mellitus cases as compared to non-diabetic individuals. So far, there are no data from diabetes research concerning the prevalence of fungi, particularly the most common genus, i.e. *Candida*, which are important components of human colon microflora.

We aimed to examine whether there are quantitative changes of *Candida* fungi in the feces of patients with T1DM and T2DM as compared to healthy controls.

**Findings:**

Overall, we included 44 diabetic patients (27 patients with T1DM and 17 with T2DM) as well as 17 healthy, non-diabetic controls. Feces and blood samples were collected from all study individuals. DNA was isolated from fecal samples and quantitative real time PCR (qPCR) was applied in order to determine the number of fungal cells. Statistical association with selected clinical and biochemical features was examined.

There was a difference in the amount of *Candida* in the feces among the three examined groups (p = 0.007). *Candida* spp. populations in T1DM and T2DM subjects were larger as compared to controls (p = 0.017 and p = 0.037, respectively). However, no difference was found between T1DM and T2DM. No association was identified between the quantity of fungi and examined patients’ characteristics, except for negative correlation with blood lipid parameters in T2DM group.

**Conclusions:**

*Candida* fungi appear to be more prevalent in the feces of patients with T1DM and T2DM. Their amount seems to be associated with serum lipids in T2DM patients. This initial finding requires further confirmation.

## Introduction

Globally, the total number of patients with both type 1 (T1DM) and type 2 diabetes (T2DM) has reached epidemic proportions. This creates enormous problems linked to health, economics and society around the world. Identification of all factors influencing the occurrence of T1DM and T2DM as well as modifying its clinical course is of great scientific and clinical importance. One of such possible factors are bacterial and fungal microbiota of the gastrointestinal tract. These microbiota influence, for example, the speed of decomposition of complex food components [1,2]. Thus, they may potentially influence pathogenesis of T1DM, as the evidence exists that processes of digestion and absorption modify the occurrence of autoagression, a major factor underlying this disease [3]. Gastrointestinal tract microbiota have also been linked with obesity, that is strongly associated with pathogenesis of T2DM [4]. Finally, the speed of digestion and absorption of carbohydrates substantially influences glucose levels in patients with diabetes [5]. Thus, determining changes in the composition of the gastrointestinal tract microbiota in the course of natural history of T1DM and T2DM could be potentially useful for developing preventive and therapeutic measures, for example through the use of probiotics. Such a use has already been described in some diseases, e.g., irritable bowel syndrome [6].

So far, some studies on changes in the bacterial microflora of the human gastrointestinal tract in the course of diabetes have been published. However, there are not enough data from diabetes research concerning fungi, particularly those entailing the most prevalent genus, *Candida*, which is an important component of human colonic microflora.

In the current study, we aimed to examine whether there are quantitative changes of *Candida* fungi in the feces of patients with T1DM and T2DM as compared to the healthy controls.

## Materials and methods

### Patients

Overall, we included 44 diabetic patients (27 patients with T1DM and 17 with T2DM) as well as 17 healthy non-diabetic controls. Their characteristics are provided in Table 1; all the subjects were white Caucasians, residents of south-eastern Poland. The patients with diabetes remained under medical care of the Department of Metabolic Diseases, University Hospital, Krakow, Poland.

**Table 1:** **Characteristics of the studied patient groups**

	**T1DM**	**T2DM**	**Control**
**Male : Female (n)**	10 : 17	7 : 10	5 : 12
	27	17	17
Age* (yrs)	35.50 &	57.43^#^	41.12
	(±7.72)	(±7.39)	(±8.49)
duration of diabetes* (yrs)	12.61^#^	9.29^#^	0.0
	(±9.04)	(±5.04)	(±0.0)
BMI* [kg/m ^**2**^ **]**	24.18 ± 3.61&	30.91 ± 4.73^#^	22.94 ± 1.4
glucose* [mmol/l]	7.17 ± 1.35^#^	7.07 ± 1.03^#^	4.73 ± 0.4
**total cholesterol [mmol/l]**	4.78 ± 0.79	4.63 ± 0.92	5.37 ± 0.53
HDL* [mmol/l]	1.60 ± 0.25&	1.05 ± 0.22^#^	1.76 ± 0.25
**LDL [mmol/l]**	2.74 ± 0.62	2.85 ± 0.83	3.21 ± 0.5
triglycerides (TG)* [mmol/l]	1.09 ± 0.45&	1.80 ± 0.58^#^	0.93 ± 0.33
HbA1c* [%]	8.90 ± 1.89^#^	9.00 ± 1.74^#^	5.44 ± 0.26

The presented data are means ± SD.

*significant differences within the three compared groups of patients (p < 0.05) – Kruskal–Wallis test.

#significant differences between the examined group of patients and the controls (p < 0.05) – Mann–Whitney *U* Test.

&significant differences between the examined groups of patients (T1DM vs.T2DM) (p < 0.05) – Mann–Whitney *U* Test.

T1DM patients had a clinical diagnosis of this type of diabetes, their insulin therapy was implemented in the first year following diagnosis and disease duration was at least 2 years. T2DM patients, in addition to a clinical diagnosis of non-insulin dependent diabetes, must have been on oral hypoglycemic agents for least 2 years following the diagnosis, their disease duration was 2 years or more. The control group consisted of apparently healthy non-diabetic subjects. Basic clinical data, such as diabetes duration (for T1DM and T2DM groups), age at the examination, weight and height were collected. We excluded all individuals who were treated with antibiotics or were using probiotic preparations during the period of 30 days before collecting the feces samples. Other exclusion criteria included confirmed infections of the gastrointestinal tract, chronic inflammatory bowel diseases, neoplasms and immunodeficiency syndromes.

The study was approved by the Jagiellonian University Bioethical Committee. Informed consent was provided by all patients.

#### Samples

Feces and blood samples were collected from all patients participating in the study. Using the blood samples, the following parameters were determined: lipid profile (HDL, LDL, total cholesterol, triglycerides), glycated hemoglobin (HbA_1c_) and serum fasting glucose levels. Feces samples were transported under deep-freezing conditions to the microbiological laboratory where DNA was extracted according to the previously described procedure [7].

#### Quantitative real time PCR (qPCR)

**Figure 1:**
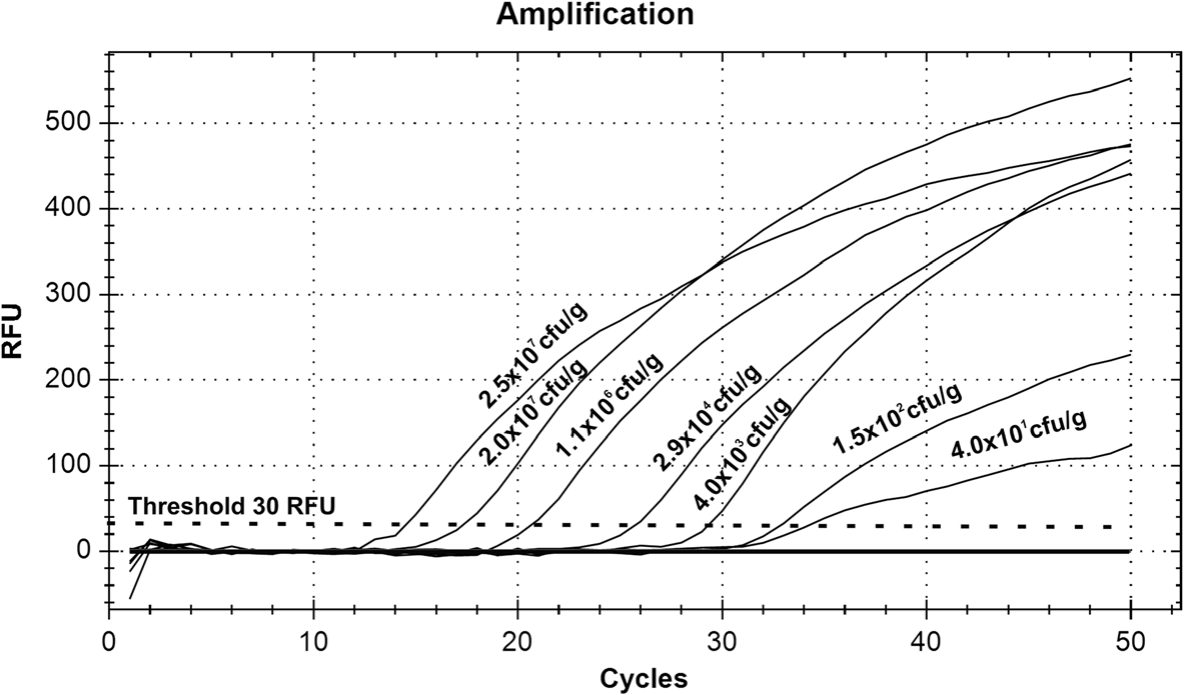
**Relative fluorescence units is the increase in reporter FAM dye intensity relative to the passive internal reference dye.** The amount of *C. albicans* ATCC10231 DNA in each sample is shown in the chart. The threshold fluorescence, or level at which the threshold cycle was determined, is shown.

**Figure 2:**
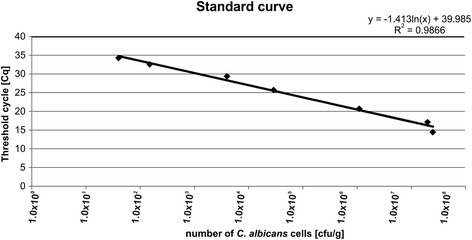
**Standard curve was generated from the amplification plot in Figure** **1** **.** y = -1.413ln(x) + 39.985; correlation coefficient (R^2^) = 0.9866. Threshold cycle was the cycle number when the threshold fluorescence was reached.

*Candida* spp. in the fecal samples were quantified by qPCR. To detect *Candida* DNA, JumpStart Taq ReadyMix for Quantitative PCR kit (Sigma), TaqMan probe (FAM-5′-TTAACCTACTAAATAGTGCTGCTAGC-BHQ1-3′) and pairs of specific primers (5′-TTGGTGGAGTGATTTGTCTGCT-3′; 5′-TCTAAGGGCATCACAGACCTG-3′) were used (Genomed) [8]. A standard curve was prepared. DNA from *C. albicans* ATCC10231 was added in serial dilutions corresponding to 10^1^ to 10^7^ cells to a series of qPCRs. The reactions were carried out on a CFX96 thermocycler (BioRad). The results are shown in (Figure 1). To determine the number of *Candida* cells, the fluorescent signals detected in DNA feces samples (in duplicate) in the linear range of the assay were averaged and compared to the standard curve (Figure 2).

#### Statistical analysis

The comparisons were made using the Kruskal–Wallis Test to describe the total distribution (in patient groups and the controls) of the studied parameters, including the number of *Candida* cells. The Mann–Whitney *U* test was used to analyze differences between the T1DM and T2DM patient groups and controls. Correlations between numbers of *Candida* spp. and age, diabetes duration, BMI, as well as biochemical features, in the examined groups were assessed using the Spearman Rho correlation coefficient. The value *p* < 0.05 was regarded as the threshold for statistical significance. All data analysis was performed using SAS 9.1 package and SAS Enterprise Quide 3.0 (SAS Institute, USA).

## Results

Clinical characteristics of the examined study group are shown in Table 1. The presence of *Candida* DNA was assessed quantitatively by qPCR (Figures 1 and 2). The identified differences between the groups in respect to the age, T1DM duration, BMI and glycemic control were in line with the way the groups were defined.

**Figure 3:**
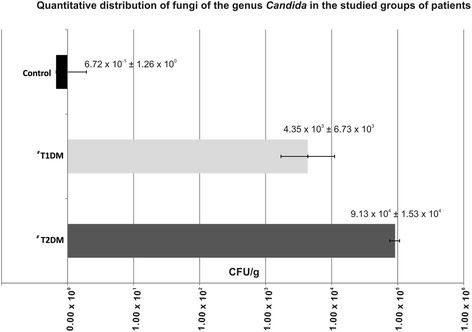
**Quantitative evaluation of fungi of the genus** ***Candida*** **in the feces of patients with T1DM, T2DM and control using qPCR method: # significant differences between the examined group of patients and the controls (p < 0.05); there was a difference in the amount of** ***Candida*** **in the feces among the three examined groups (p = 0.007).**

There was a difference in the amount of *Candida* in the feces among the three examined groups (p = 0.007). *Candida* spp. populations in T1DM and T2DM subjects were larger as compared to controls (p = 0.017 and p = 0.037, respectively). However, no difference was found between T1DM and T2DM (Figure 3).

No association was identified between the quantity of fungi and the examined characteristics within the individual study groups, except for negative correlation with blood lipid parameters: total cholesterol (ρ = -0.770, p = 0.03), HDL (ρ = -0.934, p = 0.002), LDL (ρ = -0.830, p = 0.016) and triglycerides (ρ = -0.755, p = 0.035) in T2DM patient group.

## Discussion

This is perhaps the first study wherein we e demonstrate occurrence of a larger number of *Candida* fungi in the feces of patients with diabetes as compared to the control group.

The genus *Candida* belongs to the microbiota group that is normally present in the gastrointestinal tract. There are some reports of associations between the occurrence of symptomatic infections caused by fungi of the genus *Candida* (candidiasis) and diabetes mellitus [9]. However, there is a clear lack of studies on the presence or occurrence of *Candida* spp. in the colon during the course of T1DM and T2DM in different patient populations except a lone study conducted using microbiological culture methods in children with T1DM [10]. These children were characterized by increased number of *Candida* as compared with the control non-diabetic individuals.

Our results suggest that in diabetic patients the colonization of the colon by *Candida* fungi is more prominent than in people without diabetes. In our opinion, the differences in the number of *Candida* in the studied groups are very unlikely to be associated with pathogenesis of diabetes, but are probably secondary to diabetes. One of the potential reasons could be related to an altered function of the immune system in diabetic patients with poor glycemic control, such as in our cohort. The other putative cause may be a direct effect of elevated blood glucose levels creating specific conditions for intensive fungal colonization. Although, in this study, no association was demonstrated between the level of blood glucose and the number of fungi, there was already a published study showing such a correlation [11]. However, in this earlier research, the samples comprising rectal swabs were taken only from women and analyzed with semi-quantitative evaluation of the number of *Candida* cells by microbiological culture methods [11]. Evaluation of the feces samples by qPCR method is much more reliable.

Our data suggest existence of a negative correlation between the number of *Candida* cells in the large intestines and the levels of serum lipids, such as total cholesterol, HDL, LDL and triglycerides in T2DM subjects. This is the first such observation made in humans. However, there are some studies performed *in vitro* that examined the relationships between fungi and lipids. For example, triglyceride-hydrolyzing lipase isolated from the *Candida viswanathii* fungal strain of natural origin reduced the concentration of triglycerides [12]. Another study has reported that some species of fungi of the genus *Saccharomyces* (related to *Candida*), demonstrated the ability to reduce the concentration of cholesterol in the culture medium [13]. The above findings may suggest that the genus *Candida* can also affect the levels of lipids in the colonized host. The mechanism may be related to the decomposition of these substances in the gastrointestinal tract, which reduces their absorption. Such properties have been proved in certain strains of probiotic bacteria, commonly used as dietary supplements or drugs [14,15]. These studies suggest that bacteria are capable of mediating reduction of lipid concentration in the host organism, therefore, analogous properties could potentially be attributed to at least some species of fungi comprising the colon microbiota. This claim requires further, more detailed experiments involving isolation of specific fungi from the collected fecal samples, followed by a series of *in vitro* tests, enabling identification of the strains which have the described properties. It is puzzling why we failed to demonstrate any correlation between the number of *Candida* in the large intestine of patients with T1DM and the control group and the lipid levels. Perhaps there were not enough fungal cells in the T1DM group to cause such an effect, although, differences between these groups were not significant. It is also possible that in the T2DM group there were other types of *Candida* strains capable of lipid degradation. This could have been related to higher BMI in T2DM as compared to the T1DM group and the control. A higher BMI suggests increased fat content in patients’ diet, which may influence increase in the level of digestive tract colonization by *Candida* strains capable of breaking down lipids. The obtained results are preliminary and require confirmation on a larger number of patients. The list of limitations of the current study includes single-point examination, limited sample size of the study groups and inclusion of only individuals with relatively long course of diabetes. Thus, our observations might require further confirmation.

## Conclusion

*Candida* fungi appear to be more prevalent in the feces of patients with T1DM and T2DM with poor glycemic control than in individuals without diabetes. Their amount seems inversely correlated with the serum lipids in T2DM.

## References

[1] DiBaise JK, Zhang H, Crowell MD, Krajmalnik-Brown R, Decker GA, Rittmann BE (2008). Gut microbiota and its possible relationship with obesity. Mayo Clin Proc.

[2] Hao W-L, Lee Y-K (2004). Microflora of the gastrointestinal tract: a review. Methods Mol Biol.

[3] Zipris D (2013). The interplay between the gut microbiota and the immune system in the mechanism of type 1 diabetes. Curr Opin Endocrinol Diabetes Obes.

[4] Moreno-Indias I, Cardona F, Tinahones FJ, Queipo-Ortuño MI (2014). Impact of the gut microbiota on the development of obesity and type 2 diabetes mellitus. Front Microbiol.

[5] Zhang X, Shen D, Fang Z, Jie Z, Qiu X, Zhang C, Chen Y, Ji L (2013). Human gut microbiota changes reveal the progression of glucose intolerance. PLoS One.

[6] Mach T (2006). Clinical usefulness of probiotics in inflammatory bowel diseases. J Physiol Pharmacol.

[7] Pilarczyk-Zurek M, Chmielarczyk A, Gosiewski T, Tomusiak A, Adamski P, Zwolinska-Wcislo M, Mach T, Heczko PB, Strus M (2013). Possible role of *Escherichia coli* in propagation and perpetuation of chronic inflammation in ulcerative colitis. BMC Gastroenterol.

[8] Sugita S, Kamoi K, Ogawa M, Watanabe K, Shimizu N, Mochizuki M (2012). Detection of *Candida* and *Aspergillus* species DNA using broad-range real-time PCR for fungal endophthalmitis. Graefe’s Arch Clin Exp Ophthalmol.

[9] Al Mubarak S, Robert AA, Baskaradoss JK, Al-Zoman K, Al Sohail A, Alsuwyed A, Ciancio S (2013). The prevalence of oral *Candida* infections in periodontitis patients with type 2 diabetes mellitus. J Infect Public Health.

[10] Soyucen E, Gulcan A, Aktuglu-Zeybek AC, Onal H, Kiykim E, Aydin A (2013). Differences in the gut microbiota of healthy children and those with type 1 diabetes. Pediatr Int.

[11] Nowakowska D, Kurnatowska A, Stray-Pedersen B, Wilczyński J (2004). Species distribution and influence of glycemic control on fungal infections in pregnant women with diabetes. J Infect.

[12] De Almeida AF, Tauk-Tornisielo SM, Carmona EC (2013). Acid lipase from *Candida viswanathii*: production, biochemical properties, and potential application. Biomed Res Int.

[13] Psomas EI, Fletouris DJ, Litopoulou-Tzanetaki E, Tzanetakis N (2003). Assimilation of cholesterol by yeast strains isolated from infant feces and Feta cheese. J Dairy Sci.

[14] Salaj R, Stofilová J, Soltesová A, Hertelyová Z, Hijová E, Bertková I, Strojný L, Kružliak P, Bomba A: **The effects of two*****Lactobacillus plantarum*****strains on rat lipid metabolism receiving a high fat diet.** *SciWorldJ* 2013, **ᅟ:**135142. doi:10.1155/2013/135142. eCollection 2013. 10.1155/2013/135142 PMC3891428 24470789

[15] Anandharaj M, Sivasankari B (2014). Isolation of potential probiotic *Lactobacillus oris* HMI68 from mother’s milk with cholesterol-reducing property. J Biosci Bioeng.

